# *catena*-Poly[[tetraaquanickel(II)]-μ_3_-benzene-1,3,5-tricarboxylato-3′:1:2-κ^4^ *O* ^1^:*O* ^3^,*O* ^3′^:*O* ^5^-[tetraaquanickel(II)]-μ_2_-benzene-1,3,5-tricarboxylato-2:3κ^2^ *O* ^1^:*O* ^3^-[tetraaquanickel(II)]]

**DOI:** 10.1107/S1600536809016729

**Published:** 2009-05-14

**Authors:** Shih-Chen Hsu, Pei-Hsuan Chiang, Chih-Hsien Chang, Chia-Her Lin

**Affiliations:** a Department of Chemistry, Chung-Yuan Christian University, Chung-Li 320, Taiwan

## Abstract

The microwave solvothermal reaction of nickel nitrate with trimesic acid provided the title compound, [Ni_3_(BTC)_2_(H_2_O)_12_]_*n*_ (BTC = benzene-1,3,5-tricarboxyl­ate anion, C_9_H_3_O_6_), which is a metal coordination polymer composed of one-dimensional zigzag chains. The crystal under investigation was ramecically twinned with an approximate twin domain ratio of 1:1. In the asymmetric unit, there are two types of Ni atoms. One of the NiO_6_ groups (2 symmetry) is coordinated to only one carboxyl­ate group and thus terminal, the other is bridging, forming the coordination polymer. The extended chains are connected by the organic BTC anions *via μ*_2_-linkages. O—H⋯O hydrogen bonds and π–π inter­actions between the chains [centroid–centroid distance 3.58 (1) Å] induce the complex to mimic a three-dimensional structure.

## Related literature

For background information on the solvothermal synthesis of coordination polymers with organic carboxyl­ate ligands, see: Kitagawa *et al.* (2004 ▶).
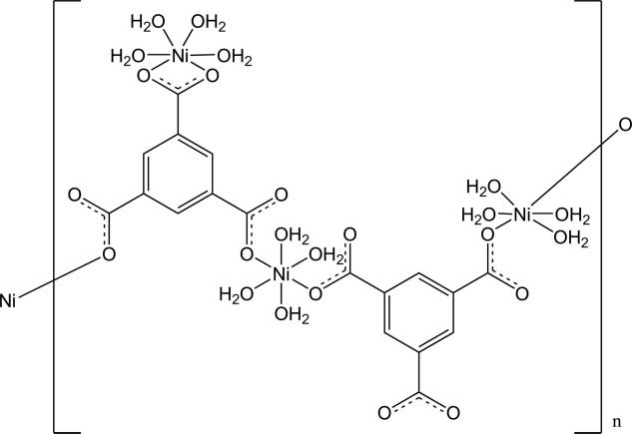


## Experimental

### 

#### Crystal data

[Ni_3_(C_9_H_3_O_6_)_2_(H_2_O)_12_]
*M* *_r_* = 806.49
Monoclinic, 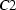

*a* = 17.3394 (10) Å
*b* = 12.8724 (7) Å
*c* = 6.5462 (3) Å
β = 111.609 (2)°
*V* = 1358.42 (12) Å^3^
*Z* = 2
Mo *K*α radiation
μ = 2.17 mm^−1^
*T* = 295 K
0.25 × 0.18 × 0.15 mm

#### Data collection

Bruker APEXII CCD diffractometer
Absorption correction: multi-scan (*SADABS*; Bruker, 2008 ▶) *T*_min_ = 0.613, *T*_max_ = 0.737
6798 measured reflections
3299 independent reflections
3156 reflections with *I* > 2σ(*I*)
*R*_int_ = 0.046

#### Refinement

*R*[*F* ^2^ > 2σ(*F* ^2^)] = 0.028
*wR*(*F* ^2^) = 0.068
*S* = 1.07
3299 reflections
208 parameters
1 restraint
H-atom parameters constrained
Δρ_max_ = 0.48 e Å^−3^
Δρ_min_ = −0.38 e Å^−3^
Absolute structure: Flack (1983 ▶), 1531 Friedel pairs
Flack parameter: 0.549 (12)

### 

Data collection: *APEX2* (Bruker, 2008 ▶); cell refinement: *SAINT* (Bruker, 2008 ▶); data reduction: *SAINT*; program(s) used to solve structure: *SHELXS97* (Sheldrick, 2008 ▶); program(s) used to refine structure: *SHELXL97* (Sheldrick, 2008 ▶); molecular graphics: *SHELXTL* (Sheldrick, 2008 ▶); software used to prepare material for publication: *SHELXTL*.

## Supplementary Material

Crystal structure: contains datablocks I, global. DOI: 10.1107/S1600536809016729/zl2190sup1.cif

Structure factors: contains datablocks I. DOI: 10.1107/S1600536809016729/zl2190Isup2.hkl

Additional supplementary materials:  crystallographic information; 3D view; checkCIF report

## Figures and Tables

**Table 1:** Hydrogen-bond geometry (Å, °)

*D*—H⋯*A*	*D*—H	H⋯*A*	*D*⋯*A*	*D*—H⋯*A*
O9—H9*B*⋯O10^i^	0.87	1.90	2.767 (3)	172
O9—H9*A*⋯O12^ii^	0.81	2.33	3.102 (3)	160
O6—H6*B*⋯O1^iii^	0.97	1.98	2.942 (3)	173
O6—H6*A*⋯O11^iv^	0.85	1.83	2.683 (3)	173
O5—H5*B*⋯O12^iv^	0.92	1.95	2.825 (3)	158
O5—H5*A*⋯O11	0.81	1.79	2.559 (3)	156
O4—H4*C*⋯O1^i^	0.92	1.97	2.870 (3)	167
O4—H4*B*⋯O10	0.91	1.77	2.617 (3)	154
O3—H3*B*⋯O12^v^	0.94	1.97	2.907 (3)	171
O3—H3*A*⋯O4^vi^	0.94	2.03	2.917 (3)	156
O2—H2*B*⋯O5^vii^	0.83	1.81	2.638 (3)	173
O2—H2*A*⋯O12^viii^	0.85	2.02	2.861 (4)	171

Symmetry codes: (i) 
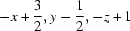
; (ii) 
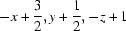
; (iii) 
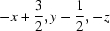
; (iv) 
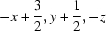
; (v) 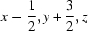; (vi) 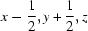; (vii) 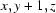; (viii) 
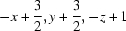
.

**Fig. 1.**
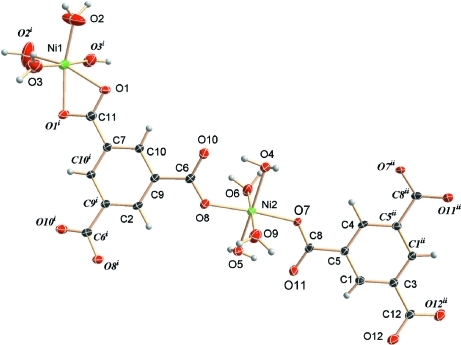
A view of the title compound, showing 50% probability displacement ellipsoids. [symmetry codes: (i) -x+1, y, -z; (ii) -x+2, y, -z+1].

**Fig. 2.**
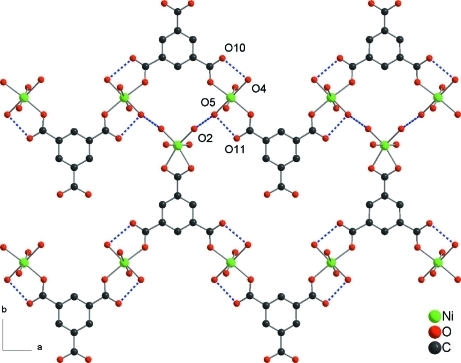
The zigzag chains of the title compound with hydrogen bonding (blue dashed lines, H atoms are omitted).

**Fig. 3.**
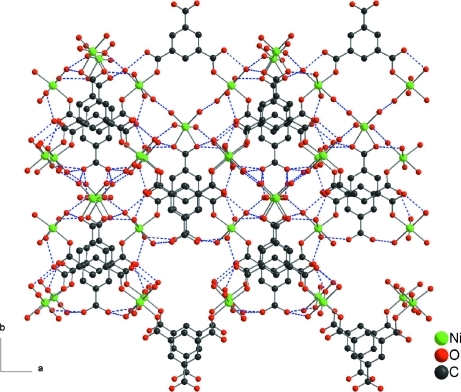
The packing diagram of zigzag chains with hydrogen bonding (blue dashed lines, H atoms are omitted).

**Fig. 4.**
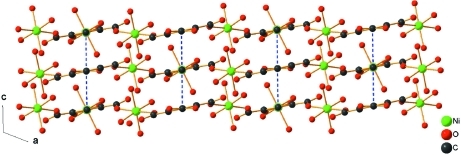
The side view of the layers with the pi-pi interactions (blue dashed lines, H atoms are omitted).

## supplementary crystallographic information

### Comment

The synthesis of coordination polymers has been a subject of intense research owing to their interesting structural chemistry and potential applications in gas storage, separation, catalysis, magnetism, and luminescence. A large number of these materials have been synthesized by solvothermal reactions with organic carboxyl acids (Kitagawa *et al.* 2004). The coordination polymers commonly adopt three-dimensional, two-dimensional, and one-dimensional structures *via* employed metal ions as connectors and rigid or flexible organic ligands as linkers. As a further study of such a complex, we report here the structure of the title compound, a nickel coordination polymer with one dimensional zigzag chains.

The crystal structure analysis of the title compound reveals the structure to be composed of zigzag chains. The compound has a non-centrosymmetric *C*2 space group and the crystal under investigation was twinned with a Flack parameter of 0.549 (12). The asymmetric unit contains two types of NiO_6_ groups (Fig. 1). The group of Ni1 is terminal and the metal atom is coordinated in a bidentate fashion to one carboxylate ligand and to four water oxygen atoms. The other nickel atom, Ni2, is coordinated in the axial positions by two monodenate carboxylate groups, and by four water molecules in the equatorial positions. All Ni–O bond lengths range from 2.021 (3) to 2.102 (3) Å. The BTC anions also have two types of coordination modes towards the NiO_6_ groups. One of the BTC bridges between two Ni2 atoms *via* two of its carboxylate groups. The third carboxylate is protonated and not metal coordinated. The other BTC ligand bridges *via* two of its carboxylates between two Ni2 atoms. Its third carboxylate group coordinates to a Ni1 atom. The one-dimensional chains thus formed are further linked with each other by hydrogen bonds and π-π interactions to form a layered structure. Hydrogen bonding interactions between coordination waters are O2–H2B···O5^ix^, O3–H3A···O4^viii^, O4–H4B···O10, O4–H4C···O1^iii^, O5–H5A···O11, O6–H6A···O11^vi^, O6–H6B···O1^v^, and O9–H9B···O10^iii^ (Fig. 2). The uncoordinated carboxylate group is involved in hydrogen bonding *via* O2–H2A···O12^x^, O3–H3B···O12^vii^, O5–H5B···O12^vi^, and O9–H9A···O12^iv^ between nearby layers (Fig. 3, see table 1 for numerical values and symmetry operators). π-π stacking interactions are found between aromatic rings made up of C1 to C5, C1^ii^ and C5^ii^, and the ring defined by C2, C7, C9, C10, C10^i^ and C9^i^ (symmetry operators: (i) -*x*+1, *y*, -*z*; (ii) -*x*+2, *y*, -*z*+1). The centroid to centroid distance between Cg1 and Cg2^ix^ defined by the two rings is 3.58 (1) Å. The rings are slipped against each other, and the approximate interplanar distance is 3.27 Å (as defined by the distance of carbon atom C4 and Cg2^ix^ (symmetry operator: (ix) 1/2+*x*, -1/2+*y*, *z*). These π-π interactions connect nearby layers with each other (Fig. 4).

### Experimental

The title complex was obtained from the reaction of 1,3,5-benzenetricarboxylic acid (C_9_H_6_O_6_, H_3_BTC, 0.421 g, 2 mmol), Ni(NO_3_)_2_.6H_2_O (0.8724 g, 3 mmol), ethanol (5.0 ml) and H_2_O (5.0 ml) with pH value of 2.15. The reaction mixture was heated to 453 K for 20 minutes using a microwave output power of 400 W. The title compound in the form of green crystals was collected in a yield of 0.0979 g (12.2%, based on carboxylic acid reagent).

### Refinement

The hydrogen atoms of benzene rings are placed in idealized positions and constrained to ride on their parent atoms, with C—H = 0.93 Å and *U*_iso_(H) = 1.2 *U*_eq_(C). The hydrogen atoms of water molecules were found in difference Fourier maps and were refined using distance constraints with O—H = 0.81 to 0.96 Å with *U*_iso_(H) = 1.2 *U*_eq_(O). Friedel pairs were not merged prior to refinement. The value of the Flack parameter and its standard uncertainty were determined by full-matrix least-squares refinement using the TWIN/BASF commands in the *SHELXTL* program. It refined to 0.55 (1).

### Crystal data

**Table:**

[Ni_3_(C_9_H_3_O_6_)_2_(H_2_O)_12_]	*F*(000) = 828
*M**_r_* = 806.49	*D*_x_ = 1.972 Mg m^−^^3^
Monoclinic, *C*2	Mo *K*α radiation, λ = 0.71073 Å
Hall symbol: C 2y	Cell parameters from 4802 reflections
*a* = 17.3394 (10) Å	θ = 2.5–28.3°
*b* = 12.8724 (7) Å	µ = 2.17 mm^−^^1^
*c* = 6.5462 (3) Å	*T* = 295 K
β = 111.609 (2)°	Columnar, light-blue
*V* = 1358.42 (12) Å^3^	0.25 × 0.18 × 0.15 mm
*Z* = 2	

### Data collection

**Table:**

Bruker APEXII CCD diffractometer	3299 independent reflections
Radiation source: fine-focus sealed tube	3156 reflections with *I* > 2σ(*I*)
graphite	*R*_int_ = 0.046
φ and ω scans	θ_max_ = 28.4°, θ_min_ = 2.0°
Absorption correction: multi-scan (*SADABS*; Bruker, 2008)	*h* = −23→22
*T*_min_ = 0.613, *T*_max_ = 0.737	*k* = −16→17
6798 measured reflections	*l* = −7→8

### Refinement

**Table:**

Refinement on *F*^2^	Secondary atom site location: difference Fourier map
Least-squares matrix: full	Hydrogen site location: inferred from neighbouring sites
*R*[*F*^2^ > 2σ(*F*^2^)] = 0.028	H-atom parameters constrained
*wR*(*F*^2^) = 0.068	*w* = 1/[σ^2^(*F*_o_^2^) + (0.0363*P*)^2^] where *P* = (*F*_o_^2^ + 2*F*_c_^2^)/3
*S* = 1.07	(Δ/σ)_max_ = 0.001
3299 reflections	Δρ_max_ = 0.48 e Å^−^^3^
208 parameters	Δρ_min_ = −0.38 e Å^−^^3^
1 restraint	Absolute structure: Flack (1983), 1531 Friedel pairs
Primary atom site location: structure-invariant direct methods	Flack parameter: 0.549 (12)

### Special details

**Table:**

Geometry. All esds (except the esd in the dihedral angle between two l.s. planes) are estimated using the full covariance matrix. The cell esds are taken into account individually in the estimation of esds in distances, angles and torsion angles; correlations between esds in cell parameters are only used when they are defined by crystal symmetry. An approximate (isotropic) treatment of cell esds is used for estimating esds involving l.s. planes.
Refinement. Refinement of *F*^2^ against ALL reflections. The weighted *R*-factor *wR* and goodness of fit *S* are based on *F*^2^, conventional *R*-factors *R* are based on *F*, with *F* set to zero for negative *F*^2^. The threshold expression of *F*^2^ > σ(*F*^2^) is used only for calculating *R*-factors(gt) *etc*. and is not relevant to the choice of reflections for refinement. *R*-factors based on *F*^2^ are statistically about twice as large as those based on *F*, and *R*- factors based on ALL data will be even larger.

### Fractional atomic coordinates and isotropic or equivalent isotropic displacement parameters (Å^2^)

**Table:**

	*x*	*y*	*z*	*U*_iso_*/*U*_eq_	
Ni1	0.5000	0.68238 (4)	0.0000	0.02554 (13)	
Ni2	0.757745 (18)	−0.02765 (2)	0.22999 (5)	0.01825 (9)	
O1	0.56460 (12)	0.54287 (15)	0.1157 (3)	0.0269 (4)	
O2	0.58008 (19)	0.7828 (2)	0.1959 (5)	0.0659 (10)	
H2A	0.5729	0.8177	0.2978	0.079*	
H2B	0.6113	0.8141	0.1460	0.079*	
O3	0.44433 (15)	0.68727 (18)	0.2333 (4)	0.0396 (5)	
H3A	0.4101	0.6382	0.2657	0.047*	
H3B	0.4382	0.7546	0.2822	0.047*	
O4	0.83989 (12)	0.08047 (16)	0.4286 (3)	0.0259 (4)	
H4B	0.8029	0.1274	0.4439	0.031*	
H4C	0.8708	0.0577	0.5683	0.031*	
O5	0.67707 (12)	−0.13068 (15)	0.0142 (3)	0.0229 (4)	
H5A	0.7035	−0.1838	0.0246	0.028*	
H5B	0.6543	−0.1191	−0.1354	0.028*	
O6	0.77890 (12)	0.03467 (16)	−0.0377 (3)	0.0316 (5)	
H6A	0.7595	0.0949	−0.0829	0.038*	
H6B	0.8290	0.0322	−0.0698	0.038*	
O7	0.85264 (11)	−0.12996 (14)	0.3010 (3)	0.0223 (4)	
O8	0.65774 (12)	0.06587 (15)	0.1685 (3)	0.0237 (4)	
O9	0.73419 (13)	−0.09194 (17)	0.4944 (3)	0.0299 (5)	
H9A	0.6945	−0.0832	0.5292	0.036*	
H9B	0.7453	−0.1563	0.5346	0.036*	
O10	0.71601 (12)	0.21051 (15)	0.3445 (3)	0.0271 (4)	
O11	0.78560 (12)	−0.27492 (14)	0.1508 (3)	0.0253 (4)	
O12	0.93126 (13)	−0.61400 (16)	0.4291 (4)	0.0333 (5)	
C1	0.92709 (16)	−0.3961 (2)	0.3927 (4)	0.0156 (5)	
H1A	0.8781	−0.4324	0.3216	0.019*	
C2	0.5000	0.1625 (3)	0.0000	0.0159 (7)	
H2C	0.5000	0.0903	0.0000	0.019*	
C3	1.0000	−0.4499 (3)	0.5000	0.0157 (7)	
C4	1.0000	−0.2341 (3)	0.5000	0.0158 (6)	
H4A	1.0000	−0.1618	0.5000	0.019*	
C5	0.92667 (15)	−0.28782 (19)	0.3907 (4)	0.0149 (5)	
C6	0.65536 (16)	0.1604 (2)	0.2156 (4)	0.0178 (5)	
C7	0.5000	0.3778 (3)	0.0000	0.0163 (7)	
C8	0.84852 (15)	−0.2270 (2)	0.2722 (4)	0.0159 (5)	
C9	0.57403 (16)	0.2158 (2)	0.1077 (4)	0.0159 (5)	
C10	0.57306 (15)	0.3237 (2)	0.1084 (4)	0.0167 (5)	
H10A	0.6218	0.3601	0.1821	0.020*	
C11	0.5000	0.4925 (3)	0.0000	0.0204 (8)	
C12	1.0000	−0.5673 (3)	0.5000	0.0211 (8)	

### Atomic displacement parameters (Å^2^)

**Table:**

	*U*^11^	*U*^22^	*U*^33^	*U*^12^	*U*^13^	*U*^23^
Ni1	0.0250 (3)	0.0154 (2)	0.0335 (3)	0.000	0.0076 (2)	0.000
Ni2	0.01496 (14)	0.01230 (14)	0.02472 (16)	0.00137 (13)	0.00407 (11)	0.00049 (13)
O1	0.0201 (10)	0.0127 (9)	0.0360 (11)	−0.0018 (8)	−0.0037 (9)	−0.0020 (8)
O2	0.081 (2)	0.065 (2)	0.0751 (18)	−0.0546 (18)	0.0557 (17)	−0.0443 (17)
O3	0.0483 (14)	0.0264 (11)	0.0539 (13)	−0.0045 (11)	0.0305 (11)	−0.0004 (11)
O4	0.0207 (10)	0.0182 (10)	0.0317 (11)	0.0019 (8)	0.0013 (9)	−0.0023 (9)
O5	0.0204 (9)	0.0168 (10)	0.0260 (9)	0.0023 (7)	0.0019 (8)	−0.0001 (8)
O6	0.0284 (11)	0.0259 (11)	0.0450 (12)	0.0085 (9)	0.0187 (10)	0.0130 (9)
O7	0.0154 (9)	0.0131 (9)	0.0353 (11)	0.0028 (7)	0.0057 (8)	0.0000 (8)
O8	0.0203 (9)	0.0134 (9)	0.0345 (11)	0.0048 (7)	0.0068 (9)	−0.0024 (8)
O9	0.0294 (11)	0.0268 (11)	0.0337 (10)	0.0060 (9)	0.0120 (9)	0.0091 (8)
O10	0.0203 (10)	0.0203 (10)	0.0335 (10)	0.0044 (8)	0.0013 (9)	−0.0056 (8)
O11	0.0179 (9)	0.0163 (9)	0.0327 (10)	0.0029 (7)	−0.0014 (8)	−0.0047 (8)
O12	0.0333 (12)	0.0145 (10)	0.0376 (12)	−0.0044 (9)	−0.0040 (10)	0.0022 (9)
C1	0.0168 (12)	0.0127 (12)	0.0161 (12)	−0.0027 (10)	0.0045 (10)	−0.0025 (9)
C2	0.0224 (17)	0.0081 (16)	0.0183 (16)	0.000	0.0089 (14)	0.000
C3	0.0200 (17)	0.0096 (16)	0.0155 (16)	0.000	0.0039 (14)	0.000
C4	0.0202 (16)	0.0100 (15)	0.0184 (15)	0.000	0.0085 (13)	0.000
C5	0.0142 (11)	0.0137 (11)	0.0157 (11)	0.0025 (8)	0.0042 (9)	0.0000 (8)
C6	0.0193 (12)	0.0174 (13)	0.0171 (11)	0.0021 (10)	0.0073 (10)	0.0016 (9)
C7	0.0194 (17)	0.0130 (16)	0.0167 (16)	0.000	0.0068 (14)	0.000
C8	0.0159 (11)	0.0145 (12)	0.0187 (11)	0.0010 (9)	0.0081 (9)	−0.0004 (9)
C9	0.0183 (12)	0.0134 (11)	0.0178 (12)	0.0025 (9)	0.0087 (10)	0.0006 (9)
C10	0.0144 (11)	0.0156 (12)	0.0194 (12)	−0.0013 (9)	0.0052 (10)	0.0002 (9)
C11	0.0188 (16)	0.016 (2)	0.0235 (17)	0.000	0.0036 (14)	0.000
C12	0.029 (2)	0.0099 (17)	0.0175 (17)	0.000	0.0009 (15)	0.000

### Geometric parameters (Å, °)

**Table:**

Ni1—O2^i^	1.983 (3)	O9—H9A	0.8087
Ni1—O2	1.983 (3)	O9—H9B	0.8686
Ni1—O3^i^	2.087 (2)	O10—C6	1.257 (3)
Ni1—O3	2.087 (2)	O11—C8	1.250 (3)
Ni1—O1^i^	2.1043 (19)	O12—C12	1.261 (3)
Ni1—O1	2.1043 (19)	C1—C3	1.385 (3)
Ni1—C11	2.445 (4)	C1—C5	1.393 (4)
Ni2—O7	2.0235 (18)	C1—H1A	0.9300
Ni2—O8	2.026 (2)	C2—C9	1.396 (3)
Ni2—O5	2.0630 (19)	C2—C9^i^	1.396 (3)
Ni2—O4	2.0716 (19)	C2—H2C	0.9300
Ni2—O6	2.0787 (19)	C3—C1^ii^	1.385 (3)
Ni2—O9	2.090 (2)	C3—C12	1.512 (5)
O1—C11	1.275 (3)	C4—C5	1.392 (3)
O2—H2A	0.8508	C4—C5^ii^	1.392 (3)
O2—H2B	0.8329	C4—H4A	0.9300
O3—H3A	0.9431	C5—C8	1.509 (3)
O3—H3B	0.9439	C6—C9	1.504 (3)
O4—H4B	0.9132	C7—C10	1.390 (3)
O4—H4C	0.9216	C7—C10^i^	1.390 (3)
O5—H5A	0.8116	C7—C11	1.476 (5)
O5—H5B	0.9233	C9—C10	1.390 (3)
O6—H6A	0.8533	C10—H10A	0.9300
O6—H6B	0.9664	C11—O1^i^	1.275 (3)
O7—C8	1.261 (3)	C12—O12^ii^	1.261 (3)
O8—C6	1.260 (3)		
			
O2^i^—Ni1—O2	98.7 (2)	H5A—O5—H5B	103.5
O2^i^—Ni1—O3^i^	84.80 (10)	Ni2—O6—H6A	118.3
O2—Ni1—O3^i^	92.94 (10)	Ni2—O6—H6B	128.5
O2^i^—Ni1—O3	92.94 (10)	H6A—O6—H6B	103.1
O2—Ni1—O3	84.80 (10)	C8—O7—Ni2	127.85 (17)
O3^i^—Ni1—O3	176.54 (13)	C6—O8—Ni2	128.92 (18)
O2^i^—Ni1—O1^i^	99.80 (12)	Ni2—O9—H9A	128.6
O2—Ni1—O1^i^	160.42 (11)	Ni2—O9—H9B	122.6
O3^i^—Ni1—O1^i^	95.20 (9)	H9A—O9—H9B	99.5
O3—Ni1—O1^i^	87.75 (9)	C3—C1—C5	120.4 (2)
O2^i^—Ni1—O1	160.42 (11)	C3—C1—H1A	119.8
O2—Ni1—O1	99.80 (12)	C5—C1—H1A	119.8
O3^i^—Ni1—O1	87.75 (9)	C9—C2—C9^i^	121.2 (3)
O3—Ni1—O1	95.20 (9)	C9—C2—H2C	119.4
O1^i^—Ni1—O1	62.84 (10)	C9^i^—C2—H2C	119.4
O2^i^—Ni1—C11	130.67 (11)	C1^ii^—C3—C1	119.9 (3)
O2—Ni1—C11	130.67 (11)	C1^ii^—C3—C12	120.03 (16)
O3^i^—Ni1—C11	91.73 (7)	C1—C3—C12	120.03 (16)
O3—Ni1—C11	91.73 (7)	C5—C4—C5^ii^	120.4 (3)
O1^i^—Ni1—C11	31.42 (5)	C5—C4—H4A	119.8
O1—Ni1—C11	31.42 (5)	C5^ii^—C4—H4A	119.8
O7—Ni2—O8	175.21 (9)	C4—C5—C1	119.4 (2)
O7—Ni2—O5	91.60 (7)	C4—C5—C8	118.9 (2)
O8—Ni2—O5	86.05 (8)	C1—C5—C8	121.6 (2)
O7—Ni2—O4	88.86 (8)	O10—C6—O8	124.4 (2)
O8—Ni2—O4	93.77 (8)	O10—C6—C9	118.7 (2)
O5—Ni2—O4	176.11 (9)	O8—C6—C9	116.9 (2)
O7—Ni2—O6	93.66 (8)	C10—C7—C10^i^	119.9 (3)
O8—Ni2—O6	90.41 (8)	C10—C7—C11	120.07 (17)
O5—Ni2—O6	87.51 (8)	C10^i^—C7—C11	120.07 (17)
O4—Ni2—O6	88.61 (8)	O11—C8—O7	124.9 (2)
O7—Ni2—O9	86.70 (8)	O11—C8—C5	118.6 (2)
O8—Ni2—O9	89.18 (8)	O7—C8—C5	116.5 (2)
O5—Ni2—O9	91.29 (8)	C10—C9—C2	118.9 (2)
O4—Ni2—O9	92.59 (8)	C10—C9—C6	118.9 (2)
O6—Ni2—O9	178.76 (9)	C2—C9—C6	122.2 (2)
C11—O1—Ni1	89.19 (17)	C9—C10—C7	120.6 (3)
Ni1—O2—H2A	125.6	C9—C10—H10A	119.7
Ni1—O2—H2B	117.6	C7—C10—H10A	119.7
H2A—O2—H2B	110.8	O1—C11—O1^i^	118.8 (3)
Ni1—O3—H3A	130.1	O1—C11—C7	120.61 (16)
Ni1—O3—H3B	114.6	O1^i^—C11—C7	120.61 (16)
H3A—O3—H3B	112.3	O1—C11—Ni1	59.39 (16)
Ni2—O4—H4B	99.5	O1^i^—C11—Ni1	59.39 (16)
Ni2—O4—H4C	115.0	C7—C11—Ni1	180.0
H4B—O4—H4C	106.1	O12^ii^—C12—O12	123.1 (4)
Ni2—O5—H5A	105.4	O12^ii^—C12—C3	118.45 (18)
Ni2—O5—H5B	122.7	O12—C12—C3	118.45 (18)

Symmetry codes: (i) −*x*+1, *y*, −*z*; (ii) −*x*+2, *y*, −*z*+1.

### Hydrogen-bond geometry (Å, °)

**Table:**

*D*—H···*A*	*D*—H	H···*A*	*D*···*A*	*D*—H···*A*
O9—H9B···O10^iii^	0.87	1.90	2.767 (3)	172
O9—H9A···O12^iv^	0.81	2.33	3.102 (3)	160
O6—H6B···O1^v^	0.97	1.98	2.942 (3)	173
O6—H6A···O11^vi^	0.85	1.83	2.683 (3)	173
O5—H5B···O12^vi^	0.92	1.95	2.825 (3)	158
O5—H5A···O11	0.81	1.79	2.559 (3)	156
O4—H4C···O1^iii^	0.92	1.97	2.870 (3)	167
O4—H4B···O10	0.91	1.77	2.617 (3)	154
O3—H3B···O12^vii^	0.94	1.97	2.907 (3)	171
O3—H3A···O4^viii^	0.94	2.03	2.917 (3)	156
O2—H2B···O5^ix^	0.83	1.81	2.638 (3)	173
O2—H2A···O12^x^	0.85	2.02	2.861 (4)	171

Symmetry codes: (iii) −*x*+3/2, *y*−1/2, −*z*+1; (iv) −*x*+3/2, *y*+1/2, −*z*+1; (v) −*x*+3/2, *y*−1/2, −*z*; (vi) −*x*+3/2, *y*+1/2, −*z*; (vii) *x*−1/2, *y*+3/2, *z*; (viii) *x*−1/2, *y*+1/2, *z*; (ix) *x*, *y*+1, *z*; (x) −*x*+3/2, *y*+3/2, −*z*+1.
